# Shapley Idioms: Analysing BERT Sentence Embeddings for General Idiom Token Identification

**DOI:** 10.3389/frai.2022.813967

**Published:** 2022-03-14

**Authors:** Vasudevan Nedumpozhimana, Filip Klubička, John D. Kelleher

**Affiliations:** ADAPT Centre, Technological University Dublin, Dublin, Ireland

**Keywords:** language model, semantics, idiom token identification, BERT, Shapley value

## Abstract

This article examines the basis of Natural Language Understanding of transformer based language models, such as BERT. It does this through a case study on idiom token classification. We use idiom token identification as a basis for our analysis because of the variety of information types that have previously been explored in the literature for this task, including: topic, lexical, and syntactic features. This variety of relevant information types means that the task of idiom token identification enables us to explore the forms of linguistic information that a BERT language model captures and encodes in its representations. The core of this article presents three experiments. The first experiment analyzes the effectiveness of BERT sentence embeddings for creating a general idiom token identification model and the results indicate that the BERT sentence embeddings outperform Skip-Thought. In the second and third experiment we use the game theory concept of Shapley Values to rank the usefulness of individual idiomatic expressions for model training and use this ranking to analyse the type of information that the model finds useful. We find that a combination of idiom-intrinsic and topic-based properties contribute to an expression's usefulness in idiom token identification. Overall our results indicate that BERT efficiently encodes a variety of information from topic, through lexical and syntactic information. Based on these results we argue that notwithstanding recent criticisms of language model based semantics, the ability of BERT to efficiently encode a variety of linguistic information types does represent a significant step forward in natural language understanding.

## 1. Introduction

The last 5 years of natural language processing research has been a record of remarkable progress in the state-of-the-art across a range of tasks (Wang et al., 2019). This progress has primarily been driven by combining distributional semantics, distributed representations, and deep learning methods for training large neural language models (Kelleher, 2019). This is most evident in the success of Transformer based models (Vaswani et al., 2017), the best known being BERT (Devlin et al., 2018). The dominance of BERT (and other large language models) within NLP has led to some researchers arguing for a reappraisal of the fields direction in terms of reconsidering the extent to which distributional analysis can provide a basis for real natural language understanding (Bender and Koller, 2020). This criticism of distributional semantics and large language models (such as BERT) is particularly pertinent because although there are a large numbers of experimental results that demonstrate the effectiveness of this approach there is less clarity with regards to why these systems work so well.

One approach to understanding the linguistic capacity of BERT, and other models that use distributed representations, is to probe the models performance on a variety of tasks (Conneau et al., 2018). The standard approach to probing BERT's capacity to identify and encode the presence of a linguistic property in a sentence is to use sentence embeddings generated by BERT to train a classifier to predict whether the property is present in a sentence. This approach to probing rests on the assumption that the classifier's success at a task indicates to what extent BERT encodes information on the linguistic property the classifier was trained to identify. There is a large body of existing work that is focused on probing BERT representations. This work spans research on syntactic structures of various kinds, such as parts of speech, syntactic chunks, and roles (Liu et al., 2019) and subject-predicate agreement (van Schijndel et al., 2019); and semantic knowledge, such as semantic roles (Ettinger, 2020), and entity types and relations (Tenney et al., 2019); and we direct the reader to Rogers et al. (2020) for a comprehensive recent overview of this probing work. Within the body of work on probing BERT's linguistic capacity the most closely related work to our own is the work reported in Nedumpozhimana and Kelleher (2021) on exploring BERT's ability to identify the presence of an idiom in a sentence. In this article, we extend the analysis of BERT's embeddings to act as a basis for a general idiom token identification model by benchmarking BERT's performance on this task against a non-contextual embeddings (namely Skip-Thought vectors), and analysing the types of information (syntactic and lexical fixedness, topic, and so on) that BERT finds most useful to be present the training data when learning this task. Furthermore, methodologically we move beyond the traditional probing framework by adapting the game theory concept of Shapley value to analyse BERT.

Idioms are commonly used in all natural languages and text genres (Sag et al., 2002), yet idiomatic expressions remain a peculiar linguistic phenomenon due to their complex characteristics, such as: discontinuity, non-compositionality, heterogeneity and syntactic variability. Consequently, automatic identification of idiomatic expressions is difficult, and it is essential for certain natural language processing applications such as machine translation (Villavicencio et al., 2005; Salton et al., 2014) and sentiment analysis (Williams et al., 2015; Spasic et al., 2017). What makes the task even more challenging is the fact that idiomatic expressions are an open set and new ones can emerge at any time. This makes creating an exhaustive list of idiomatic expressions for any language theoretically impossible (Fazly et al., 2009). Furthermore, not all occurrences of idiomatic word combinations need to be idiomatic–in certain contexts an idiom can be used in its literal, rather than figurative sense. For example, both the sentences “If you blow your top, they'll nick you double quick.” and “He blew the top of his tea and sipped it.” contain the idiomatic expression “blow top” but only in the first sentence is used it in its idiomatic sense. Studies show that literal usage of idiomatic expressions is not uncommon, and disambiguating literal and idiomatic usage of an idiomatic expression is not at all straightforward (Fazly et al., 2009; Peng et al., 2014; Salton et al., 2016).

The task of distinguishing between idiomatic and non-idiomatic instances of a phrase in context is known as *idiom token identification* (Fazly et al., 2009). To date, the most common approach is to build a separate model for each expression. Though effective, these expression-specific models have narrow applicability. Aggregating individual models makes systems cumbersome, while providing limited capacity to deal with the problem of disambiguation. Furthermore, an ensemble of expression specific models does not at all address the problem of detecting unknown idiomatic expressions; as an answer to this still open problem, developing a *general idiom token identification model* would be the preferred solution. Salton et al. (2016) demonstrated the feasibility of using a sentence embedding based approach to the task of building a general idiom token identification model. Though their work was focused on building per-expression models, they also report a general classifier using Skip-Thought (Kiros et al., 2015) sentence embeddings to be as effective as the state of the art per-expression approach. However, in the intervening period more rich and advanced embedding techniques, such as BERT (Devlin et al., 2018), have since been developed and report vastly improved performance on many NLP tasks. Therefore it is worth investigating the application of these new embedding models to the problem of general idiom token identification.

In this article, we build on the work of Salton et al. (2016) and look at how well a contemporary sentence embedding model performs on the task of general idiom token identification, on the example of English Verb-Noun Idiomatic Combinations (VNICs). We confirm that distributed representations are suitable for this task, but we also go beyond improving the state of the art and perform a variety of experiments designed to investigate what types of information BERT uses for the task, and in this way we explore what information is useful for creating a general idiom token identification model.

**Contributions: (a)** we report a new state of the art for general idiom token identification, using BERT sentence embeddings; **(b)** we demonstrate that the game theory concept of Shapley values provides a basis for analysing idiomatic usage; and **(c)** we explain the strong performance of BERT embeddings in terms of their ability to model idiom-intrinsic and topic-based properties.

## 2. Related Work

Most previous work on idiom token identification deals with building separate models for each given expression, rather than a single general model that could handle all expressions. Early work focused on Japanese idioms revealed that features normally used in word sense disambiguation worked well, while idiom-specific features were not as helpful (Hashimoto and Kawahara, 2008, 2009). By contrast, concurrent work on English idioms by Fazly et al. (2009) argued that each idiomatic expression has a distinct canonical form when used idiomatically, defined in terms of local syntactic and lexical patterns, and these intrinsic properties of the expression can be leveraged for idiom token identification.

A significant body of research explored discourse and topic-based features, instead of idiom-specific ones. Approaches based on how strongly an expression is linked to the overall cohesive structure of the discourse showed that figurative language exhibits less cohesion with the surrounding context than literal language (Sporleder and Li, 2009; Li and Sporleder, 2010a,b). A related approach was also explored by framing idiomatic expressions as semantic outliers in topic models (Feldman and Peng, 2013; Peng et al., 2014), thus leveraging an idiom's incongruity with its context.

Some of the per-expression literature also describes work using distributed representations. Peng and Feldman (2017) use word embeddings to analyze the context that a particular expression is inserted in, and predict if its usage is literal or idiomatic. They report significant improvements over their previous work. Meanwhile, Salton et al. (2016) use Skip-Thought Vectors to create distributed sentence representations and show that classifiers trained on these representations have competitive performance compared with the state of the art per-expression idiom token classification.

In this article, we emphasise the merit of a general model, i.e., a single idiom token identification model that can work across multiple idioms, as well as generalise to unseen idioms. Little work has been done on such a model aside from Li and Sporleder (2010a) who, alongside building their per-expression models, also investigated general models, and found that global lexical context and discourse cohesion were the most predictive features, and (Salton et al., 2017), who demonstrated the viability of building such a model using features based on lexical fixedness. Finally, Salton et al. (2016) use distributional embeddings for their representations to build a general model: similar to their per-expression models, they use distributed sentential semantics generated by Skip-Thought to train a general classifier that can take any sentence containing a candidate expression and predict whether its usage is literal or idiomatic. To the best of our knowledge, this is the current state of the art in sentence-level general idiom token identification, and this is the approach that we base our work on.

The literature reveals a split between research on features that are intrinsic to idioms (e.g., fixedness) and more general approaches (e.g., cohesion). There is also evidence that it is possible to create general idiom token identification models using distributed representations. This motivates the question of whether embeddings encode idiom-specific information or incongruity with the context.

## 3. General Idiom Token Identification Model and Comparison With State of the Art

The task of idiom token identification is to decide given a sentence whether any of the expressions in that sentence are used idiomatically. Within the work on idiom token identification a distinction can be made between work that focused on developing models that were able to perform idiom token identification for a specific set of expressions (which are known to have an idiomatic sense associated with them, and that the model was trained to process), and work which attempted to create models that were able to identify idiomatic usage of any expression in a sentence. In this article, we use the term *general* idiom token identification to indicate this second strand of research. Our model for this general idiom token identification task is inspired by Salton et al. (2016), who demonstrate the feasibility of using Skip-Thought embeddings (Kiros et al., 2015) for general idiom token identification. We build on their work and apply the same methodology, but instead we use BERT embeddings (Devlin et al., 2018) of a candidate sentence to detect the presence of idiomatically used expressions. We used bert-base-uncased pretrained BERT model^1^ to generate a 768 dimensional embedding for each sentence in our dataset. It is calculated as the average of the final layer word embeddings of the words in the sentence. This sentence embedding is used as input to a simple Multi-Layered Perceptron (MLP) model to classify the sentence embedding as idiomatic or non-idiomatic. The model has one hidden layer of 100 ReLUs and an output layer with a single neuron using a logistic activation function^2^.

Our model differs from the model by Salton et al. (2016) both in terms of the embeddings used (BERT rather than Skip-Thought) and the classifier (MLP rather than SVM). The switch in embedding type was motivated by an interest in testing whether the contextually specific embeddings generated by BERT could better capture idiomatic semantics, for example by more efficiently encoding textual cohesion. We used an MLP instead of an SVM because results from the prior literature indicate that an MLP is a suitable classifier to extract information from BERT sentence embedding (Conneau et al., 2018).

As the first stage of experimentation, we compared our general idiom token identification model (BERT MLP) with the state of the art model described by Salton et al. (2016), which used Skip-Thought sentence embeddings and a different classifier: a Linear-SVM classifier (Skip-Thought LinSVM). In what follows we will use this Skip-Thought LinearSVM classifer as the state-of-the-art baseline and provide both the performance results report by Salton et al. (2016) and the results we obtained using our re-implementation of this architecture. We are specifically interested in comparing the effect of using BERT versus Skip-Thought embeddings in modeling idiomatic usage. Consequently, in order to isolate the contribution of the BERT embeddings from the confounding factor of the classification algorithm, we also developed a third model, an MLP trained on Skip-Thought embeddings (Skip-Thought MLP).

In this first experiment, we trained and evaluated the performance of our BERT MLP model on the dataset used by Salton et al. (2016) to compare with the state of the art performance on general idiom token identification. For a direct comparison of the performance scores of our model with the reported state of the art performance scores we followed the procedure set-out by Salton et al. (2016) to create training dataset and testing dataset. They used the VNC-Tokens dataset^3^ (Cook et al., 2008), which is a collection of sentences containing expressions called Verb-Noun Combinations (VNC). The VNC-tokens dataset contains a total 2984 sentences with 56 different expressions. Each sentence in the dataset is labeled as *Idiomatic usage, Literal usage*, or *Unknown*. Sentences with the *Unknown* label are of little use to the general idiom token identification model, so those sentences were not considered. Out of the 56 different idiomatic expressions, 28 idiomatic expression have a skewed ratio of idiomatic usages and literal usages. Salton et al. (2016) used the remaining 28 idiomatic expressions that have a relatively balanced label distribution for their experiment. For each expression, they maintained the same ratio of idiomatic and literal usage in both train and test set. While maintaining this ratio of idiomatic usage they split the full dataset into a training set containing roughly 75% of the data and a test set containing roughly 25% of the data. By following this procedure we created a training set with 929 sentences (62.43% idiomatic usage) and a test set with 276 sentences (61.23% idiomatic usage). We trained state of the art models and our BERT MLP model on this training set and reported the performance measures on the test set. Note that this training set and test set are only used in this experiment, which compares the proposed model with state of the art model. Later in this article, we report a number of other experiments which are designed to understand the behavior of the proposed model, and for these experiments we also use the VNC-tokens dataset however the data preparation process is different (details given in relevant sections below).

We report both macro average F1 and micro average F1 scores. To calculate a macro average F1 score we first calculate an F1 score per expression and then average these F1 scores. We use macro average F1 scores because (a) Salton et al. (2016) also reported macro average F1 scores, and (b) macro averaging has the advantage that each expression will have an equal impact on the overall score, irrespective of the number of examples in the test set that contain that expression. Its disadvantage is that it does not weight each example in the test set equally. For example, in this experiment the expression *make scene* has 10 sentences whereas the expression *hit roof* only has 5 sentences in our test set. As such, the performance of a model on a test sentence with *hit roof* will have a bigger impact on the overall F1 score than the performance of the model on a sentence containing *make scene*. To account for this, we also report the micro average F1 score. In micro averaging, instead of separately calculating an F1 measure for each expression, the total counts of true positives, false positives, and false negatives from all sentences in the test set (irrespective of expression) are calculated, and the combined F1 score is calculated based on these totals. As a result, all test sentences have equal weighting toward the final F1 score. We find the micro average F1 score reliable, as each test sentence equally contributed in its calculation. We report the macro and micro average F1 scores for each model in *Seen Idiom* column of Table 1. Note this column is labeled *Seen Idiom* to mark the fact that in the creation of the training and test split for this experiment the examples for each expression in the dataset are split across the training and test set, and so the model has *seen* examples of literal and idiomatic usages of each expression in the test set during training. Importantly, however, the actual sentences in the test set do not occur in the training set. This is the experimental design followed by Salton et al. (2016). Below we also report model performance when the training and test set split is created so that no examples of usage of the expressions in the test set are included in the training set, a scenario not examined by Salton et al. (2016). It is noteworthy that in the *Seen Idiom* experimental setup our re-implementation of the Skip-Thought LinearSVM architecture of Salton et al. (2016) (row 2 of Table 1) achieved the same macro F1 score of 0.83 that was reported by Salton et al. (2016) for this architecture (row 1 of Table 1), which validates that the dataset and experiment setup we used is consistent with the reported one.

**Table 1:** Performance of General Idiom Token Identification Models: seen idiom scores are the micro and macro F1 scores on the test set composed of sentences sampled from all 28 expressions in the VNC-tokens dataset; unseen idiom scores are the average micro and macro F1 scores (along with standard deviations) across 50 runs of model training where each model is tested on a random selection of 6 idioms held out from training and trained on the sentences containing the other 22 expressions in the VNC-tokens dataset.

	**Seen Idiom**		**Unseen Idiom**			
**Model**	**Macro F1**	**Micro F1**	**Macro F1**		**Micro F1**	
			**Avg**	**Stdev**	**Avg**	**Stdev**
Skip-Thought LinearSVM (Salton et al., 2016)	0.83	-	-	-	-	-
Skip-Thought LinearSVM (re-implementation)	0.83	0.87	0.76	0.0579	0.79	0.0550
Skip-Thought MLP	0.85	0.88	0.76	0.0761	0.80	0.0726
**BERT MLP**	**0.88**	**0.90**	**0.83**	0.0690	**0.85**	0.0497

*Bold values indicate the best performance scores (highest values) of each columns*.

We have also evaluated the ability of the model to identify idiomatic usage of idiomatic expressions not in the training set. To do this we created a test set by randomly choosing 6 idiomatic expressions from 28 idiomatic expressions in the dataset used in the previous experiment. We then trained a model on the training samples with remaining 22 idiomatic expressions and evaluated the model on test samples with 6 idiomatic expressions in the test set and calculated both micro and macro F1 scores. We repeated this process 50 times to reduce the effect of bias in random selection of 6 idiomatic expressions and in the *Unseen Idiom* column of Table 1 we report the average micro and macro F1 scores across these experiments (along with the standard deviation of these results).

Examining the results we observe that MLP models consistently outperform SVM models, irrespective of input representation. Second, we see that the BERT MLP model obtains the best scores for both micro and macro average, thereby setting a new state of the art on this task. Furthermore, the performance of the BERT MLP model on Unseen Idioms is comparable with that of the Skip-Thought based models on Seen Idioms. However, perhaps most importantly, the gap in performance between BERT MLP and other models is larger in the Unseen Idiom setting than in the Seen Idiom setting, indicating that the BERT MLP model generalizes more effectively to unseen idioms than the Skip-Thought based models. Taken together these results indicate that BERT is a more effective encoder than Skip-Thought of information that is useful for the task of general idiom token identification.

## 4. Analysis of Idiomatic Expressions

In Section 2, we highlighted a distinction between prior work that focused on features that are intrinsic to idioms, such as fixedness, and contextual features, such as cohesion with the surrounding context. Furthermore, our results show that BERT embeddings are more effective than Skip-Thought for general idiom token identification. This links with the discussion in related work that BERT embeddings are designed to capture contextual information, which further indicates that contextual cohesion is an important property for the creation of a general idiom token identification model.

In the remaining parts of this article our overall goal is to better understand what types of information that are most informative of idiomatic usage. In other words, we wish to analyse the relative strength of association between each different linguistic phenomena (syntactic and/or lexical fixedness, topic, and so on) and idiomatic usage in general. One complicating factor for this analysis is that there is variation across idiomatic expressions with respect to these different forms of information. For example, the idiomatic usage of some expressions is more lexically fixed than others, whereas for other expressions idiomatic usage may be more syntactically fixed (Fazly et al., 2009). Consequently, in what follows we will first analyse which expressions in the dataset are most useful in terms of their inclusion in a training dataset contributing to the accuracy of a general idiom token identification model, and then analyse the strength of correlation between this ranking of expression usefulness with other properties of these expressions.

The experimental design we use for the analyses reported in this section involves stratified 5 fold cross validation. However, the dataset used in the previous experiment contains some idiomatic expressions that have less than 5 idiomatic or literal usage samples. Continuing to include these expressions in the dataset poses difficulties for standard stratified 5 fold cross validation. Therefore we further filtered the dataset used in the previous experiments and selected the 26 idiomatic expressions from the VNC-tokens dataset (see Table 2) which have at least 5 idiomatic usages and 5 literal usages. From each of these 26 idiomatic expressions we selected all samples with labels *Idiomatic usage* or *Literal usage*. The total number of samples and the percentage of idiomatic usages from each of these expressions are shown in the *Size* and *Ratio* columns of Table 2. For example, there are 28 example sentences containing the expression *blow top* and of these 82% are labeled as *Idiomatic usage*. Samples from these 26 idiomatic expressions have very unbalanced label distributions, as many expressions have more idiomatic usages (positive label) than literal usages. Therefore it is crucial to select a suitable performance metric for our analyses. The AUC-ROC and Mathew correlation coefficient (MCC) metrics consider both positive and negative classes and are also suitable for imbalanced datasets. We thus considered both of these as suitable evaluation metrics for the VNC dataset. Reviewing previous literature on these metrics we found an empirical comparative study by Halimu et al. (2019) which showed that both AUC-ROC and MCC are statistically consistent with each other, however, AUC-ROC is more discriminating than MCC. Therefore we selected the AUC-ROC as the metric for our analyses and used a stratified 5-fold cross validation for evaluating the performance metric of models.

**Table 2:** The 26 idiomatic expressions used in the second experiment along with summary statistics of their distribution in the dataset, their Shapley values, single expression model performance (SE), SE for seen expressions (SeenSE), SE for unseen expressions (UnseenSE), ease of prediction (Easiness), Fixedness, Topic Distributional Similarity (TDS), and Idiom Literal Divergence (ILD).

**Expressions**	**Shapley**	**SE**	**SeenSE**	**UnseenSE**	**Gap**	**Easiness**	**Size**	**Ratio**	**Fixedness**	**TDS**	**ILD**
blow top	0.0293	0.6735	0.9600	0.6709	0.2891	0.9600	28	0.8214	0.589	0.5452	1.4403
blow trumpet	0.0419	0.7800	1.0000	0.7762	0.2238	0.9750	29	0.6552	0.594	0.5401	1.4646
blow whistle	0.0534	0.8289	0.9920	0.8062	0.1858	0.9633	78	0.3462	-	0.5603	0.5798
cut figure	0.0346	0.6982	1.0000	0.6783	0.3217	0.8714	43	0.8372	0.325	0.4743	1.9835
find foot	0.0374	0.6980	0.9333	0.6897	0.2436	0.8533	53	0.9057	0.568	0.3883	0.6422
get sack	0.0283	0.6814	0.9625	0.6619	0.3006	0.9056	50	0.8600	0.450	0.4569	2.5113
get wind	0.0342	0.7187	1.0000	0.7066	0.2934	0.9000	28	0.4643	0.290	0.4172	0.5921
have word	0.0335	0.6727	0.9875	0.6502	0.3373	0.8625	91	0.8791	0.220	0.5700	0.9830
hit road	0.0365	0.7447	0.9600	0.7511	0.2089	0.9000	32	0.7813	0.414	0.5260	0.9252
hit roof	0.0418	0.7488	0.7500	0.7449	0.0051	1.0000	18	0.6111	0.414	0.5920	1.6725
hit wall	0.0458	0.7883	0.9818	0.7611	0.2208	0.9818	63	0.1111	0.322	0.5699	1.6975
hold fire	0.0401	0.8258	1.0000	0.8301	0.1699	0.9667	23	0.3043	0.277	0.4148	1.6287
kick heel	0.0349	0.7461	0.8833	0.7422	0.1411	0.9500	39	0.7949	0.718	0.4417	1.5495
lose head	0.0209	0.6032	0.8217	0.5977	0.2239	0.6567	40	0.5250	0.363	0.4341	1.5564
make face	0.0288	0.5860	0.9556	0.5536	0.4019	0.8144	41	0.6585	0.254	0.4479	0.0201
make hay	0.0345	0.7287	1.0000	0.7178	0.2822	1.0000	17	0.5294	0.406	0.5304	2.2987
make hit	0.0338	0.7175	0.9000	0.7315	0.1685	0.9000	14	0.3571	0.302	0.5007	1.7473
make mark	0.0436	0.7914	0.9667	0.7746	0.1921	0.9857	85	0.8471	0.399	0.7204	1.2531
make pile	0.0346	0.7765	1.0000	0.7610	0.2390	1.0000	25	0.3200	0.258	0.5927	2.2402
make scene	0.0280	0.6321	0.9083	0.6061	0.3022	0.8583	50	0.6000	0.276	0.5914	1.2209
pull leg	0.0353	0.7281	0.9750	0.7125	0.2625	0.9333	51	0.2157	0.302	0.4918	1.0784
pull plug	0.0466	0.8157	0.9826	0.8056	0.1770	0.9889	64	0.6875	0.431	0.7237	1.0397
pull weight	0.0449	0.7983	1.0000	0.7935	0.2065	1.0000	33	0.8182	0.478	0.4518	1.4496
see star	0.0201	0.5392	0.9818	0.4815	0.5003	0.8227	61	0.0820	0.307	0.4491	1.2043
take heart	0.0368	0.7255	0.9340	0.7063	0.2277	0.8846	81	0.7531	0.300	0.5685	0.6763
take root	0.0359	0.7082	0.9417	0.7014	0.2403	0.9542	97	0.8454	-	0.8751	3.0104
Correlation with Shapley		0.9017	0.2276	0.8619	-0.6161	0.7153	0.1711	0.0235	0.2020	0.3359	-0.1036

### 4.1. Shapley Value Analysis

We develop a method to measure the usefulness of an idiomatic expression to build a general idiom token identification model by adopting the concept of Shapley value from cooperative game theory (Shapley, 1953). In an n-player cooperative game, the Shapley value of any player is a number describing the contribution of that player to the performance of the team. The higher the Shapley value of a player the greater the contribution the player made to the team's performance. In our case, general idiom token identification is the cooperative game and the idiomatic expressions used for training are the players. In this context, the Shapley value of an expression is a measure of the information it contributes (usefulness) toward training a general idiom token identification model. In principle, to calculate the Shapley value of an idiomatic expression we should train a separate idiom token identification model for each possible subset of idioms in the set. We would then compare the performance of the models where the expression was included in the training set with the models where the expression was excluded from the training set. Idioms whose inclusion in the training set results in better model performance relative to the models where they are excluded are considered useful and have a high Shapley value. For our dataset with 26 expressions there are 2^26^ = 67, 108, 864 possible combinations of expressions, so this would entail training 2^26^ models and then to calculate the Shapley score for each expression comparing the performance of the 2^25^ models where the expression was included in the training to the performance of the 2^25^ models where the expression was not in the training set. Clearly this full Shapley value analysis is not feasible due to this combinatorial explosion of expression subset, and below we describe how we approximated this process.

One of the difficulties with the computation of Shapley values is that the execution time increases exponentially with the number of players in the game. In this case, we have 26 players and therefore the computation of a Shapley value for each of the 26 players is practically impossible (requiring 2^26^ models to be trained). So we used a randomized approximation algorithm with structured random sampling proposed by van Campen et al. (2017). Using this algorithm, the Shapley value for an expression is calculated by first randomly sampling a fixed number *n* of combinations of expressions from the 2^25^ combinations that include an expression, and then for each of these selected combination constructing a corresponding combination of expressions that does not include the expression by removing the target expression from the original combination. This process results in 2*n* combinations of expressions being selected, *n* including an expression and *n* with the expression removed. Then for each of these 2*n* expression combinations a model is trained and evaluated and then the Shapley value for an expression is the difference between the average performance of the *n* models trained with the expression in their training data and *n* models with the expression removed from the training data. In our experiments, we applied this algorithm within a 5-fold cross-validation process so that the validation set was resampled for each fold that the algorithm was applied within. Each of these 5-folds generated a Shapley value score per expression and the final Shapley score for an expression was calculated as the average Shapley score across the 5-folds. Within each fold we set *n* equal to 2 resulting in 4 models being trained per expression (2 trained with the expression and 2 trained without the expression). This process resulted in a total of 26 × 4 × 5 = 520 models being trained. Using this algorithm, approximate Shapley values for all 26 expressions were estimated and these values are listed in the second column of Table 2. The Shapley values vary from 0.0201 (interpreted as a contribution of 2% AUC-ROC toward overall performance) to 0.0534 (a contribution of 5% AUC-ROC toward overall performance), indicating that not all expressions are equally useful for the task. The expression *blow whistle* makes the greatest contribution to model performance when it is included in the training set, and *see star* makes the least contribution.

### 4.2. Why Are Some Idioms More Useful?

Our observation that there is variation in terms of the usefulness of different expressions with respect to the training of a general idiom token identification model naturally raises the question: why are some expressions more useful than others? Answering this question is both theoretically interesting, as it may provide a basis for a better understanding of properties of idioms, as well as practically relevant for selecting sentences with idiomatic expressions for inclusion in training in order to create better classification models.

Initially, we investigated whether expressions with a relatively large Shapley value (i.e., that perform well in combination with other expressions) are also more useful when used in isolation. For each individual expression we trained a separate single expression idiom token identification model (SE) by using the samples for that expression in the training folds and evaluated the model performance on test folds of all 26 expressions. We report the average AUC-ROC score from stratified 5-fold cross validation of SE models in the *SE* column of Table 2. We found a very strong positive correlation (0.9017) between SE scores and Shapley values. This is an interesting finding because the Shapley value for a given expression is a function of the change in performance of models after removing the target expression from each subsets of the training set. Consequently, it would be possible that an idiomatic expression that in isolation results in weak model performance may provide useful interactions when combined with other expressions in a training set. If these types of interactions were present in this task then we would expect the correlation between the Shapley values and the single expression performance to be lower. We, therefore, take this strong correlation to indicate that the strength of a group of expressions primarily comes from individual expressions, rather than from the interactions between groups of (potentially weak) expressions. In other words, for the task of training a general idiom token identification model a weak expression will not become strong in combination with other expressions. The implication of this is that to build a good model, we want expressions which have good individual performance.

The individual performance of an expression (i.e., the SE score) is a combination of the performance on samples of that expression (which is seen during training) and on samples from other expressions which are not seen during training. We further investigated whether the Shapley value analysis found some expressions to be useful (i.e., returned a high Shapley score for these expressions) because of their impact on helping a model to learn how to predict the idiomatic usage of these expressions themselves or the idiomatic usage of other expressions. To do this, for each expression we trained one model by using a training set containing only that single expression (as in SE above). We then calculated a seen and unseen score for each of these 26 models. The seen score for an expression's model was calculated by testing the model on a hold-out test set of examples that contained that expression. The unseen score for an expression's model was calculated by testing the model on a test-set of examples from all the remaining 25 expressions and reported in Table 2. (Note that the newly calculated seen scores and unseen scores are the decomposition of SE scores, because the SE score is calculated by testing models that are trained on individual expressions on test sets that contain all expression both the seen and the unseen expressions.) We observed that there is a strong positive correlation between Shapley values and unseen score (0.86) compared to the correlation with seen score (0.23). We also got a negative correlation (-0.62) between the Shapley values and the gap between seen and unseen, indicating that the gap between seen and unseen is less for expressions with high Shapley value. These results indicate that the Shapley value of an expression is a reflection of the usefulness of that expression for the prediction of samples containing other expressions rather than samples containing the same expression.

The Shapley value of an expression may be related to how easy it is to predict the idiomatic versus non-idiomatic usage of the expression. We trained our general idiom token identification model on all 26 expressions, but test it separately on each of the 26 individual expressions in the test set. Such per-expression scores give us an idea of how good our model is at predicting instances of that particular expression or, in turn, how easy that expression is to predict. We include this AUC-ROC score from stratified 5-fold cross validation in the *Easiness* column of Table 2. We have also tested the correlation between easiness of prediction with Shapley values and got a strong correlation with correlation coefficient 0.7153. This strong correlation suggests that useful idioms are easier to predict.

The metrics described above–Shapley values, SE and Easiness–are all performance-based metrics that give us an idea of how individual expressions behave and how useful they are during model training. We interpret them as informing us about the usefulness of individual expressions. However, we are conscious that this descriptor “*the usefulness of an individual expression*” is somewhat opaque, because it depends on a number of different factors, potentially not even related to the individual expressions themselves, but rather the subsamples of data that contain them. In order to better understand which property of an expression makes it useful, we investigate the contribution of different features of an idiomatic expressions [namely: Fixedness, Topic Distributional Similarity (TDS) and Idiom Literal Divergence (ILD)] and the properties of the data sample associated with an expression toward its usefulness. We included two topic-related features (TDS and ILD) in our analysis because topic based models are widely used in the literature on idiom token identification, and so these topic features enabled us to investigate whether topic information helps an expression be more useful.

#### 4.2.1. Fixedness

Fazly et al. (2009) proposed two forms of fixedness in their work, lexical, and syntactic fixedness. Fazly et al. (2009) illustrate the concept of lexical fixedness by comparing the idiomatic expressions *spill the beans* and *blow one's trumpet*. *Spill the beans* is given as an example of a lexically fixed expression, for example the lexical variants *spill the peas* and *spread the beans* do not have idiomatic senses associated with them. By comparison *toot one's horn* can be understood as a lexical variant of the idiom *blow one's trumpet*. So given these examples one might judge *blow one's trumpet* to be less lexically fixed as compared with *spill the beans*. Similarly the syntactic fixedness of idiomatic expressions can be illustrated by comparing the expressions *keep one's cool* with *spill the beans*. In the case of *keep one's cool*, consider the following syntactic variants: *Vasu kept cool* and *Vasu kept his cool*, both of these examples have an idiomatic sense. However, in the case of similar syntactic variation for examples with containing *spill the beans, Vasu spilled the beans* has a possible idiomatic sense but the most likely sense of *Vasu spilled his beans* is literal. These examples suggest that *keep one's cool* can retain its idiomatic sense under greater syntactic variation than *spill the beans*; i.e., *keep one's cool* is less more syntactically fixed than *spill the beans*.

Fazly et al. (2009) propose fixedness measures for each of these forms of fixedness (lexical and syntactic) that are specifically designed for verb-noun combinations. Lexical fixedness scores are calculated as the point-wise mutual information between a verb and noun as measured across a corpus. To calculate syntactic fixedness Fazly et al. (2009) define 11 syntactic patterns, involving verbs and nouns. For each verb-noun pair in the corpus they calculate the probability distribution across these 11 syntactic patterns of the verb-noun pair occurring in that pattern as measured by normalized counts of occurrences in the corpus. The syntactic fixedness of a verb-noun pair is then calculated as the divergence between it's syntactic behavior (probability distribution across the 11 predefined syntactic patterns) from the typical syntactic behavior of verb-noun pairs (the average probability distribution across the 11 syntactic patterns of all verb-noun pairs in the corpus). In this article, we use the overall fixedness which is a linear combination of both lexical fixedness and syntactic fixedness.

Fazly et al. (2009) based their approach to idiom token identification on the assumption that idiomatic usages of an expression are more likely to appear in fixed/canonical forms than non-idiomatic usages. Inspired by this work we considered fixedness as a potential feature which helps an expression to be more useful than others. Fixedness scores of each expression as per the definition of Fazly et al. (2009) were calculated and are shown in the *Fixedness* column of Table 2. We calculated their correlation with Shapley values and found a correlation coefficient of 0.2020. This weak correlation suggests that fixedness, an intrinsic property of idioms, does not define the usefulness of an expression, although it does have some influence.

#### 4.2.2. Topic Distributional Similarity

The first topic feature we used is the similarity between the topic distribution of samples belonging to one expression and the topic distribution of all samples in the entire dataset. If the topic distribution of an expression is similar to the topic distribution of the entire dataset, then that expression has maximum topical information, and therefore it can be more useful. In other words, training only on a sample of this one expression provides enough variation in topics to be representative of the topic distribution in the entire dataset that includes all expressions.

To calculate TDS we first trained a Latent Semantic Indexing (LSI) topic model^4^ on samples from the VNC dataset. LSI is an unsupervised topic-modeling approach based on the distributional hypothesis (Deerwester et al., 1990). Then for each of the 26 expressions, we calculated the KL-divergence between the topic distribution of that expression and topic distribution of all 26 idiomatic expressions. The inverse of KL-divergence is reported as the distributional similarity in the *TDS* column of Table 2. We calculated its correlation with Shapley values and found a correlation coefficient of 0.3359. This correlation is also weak but significantly better than the correlation between Shapley and fixedness, suggesting that TDS has more influence on an idiom's usefulness, but still does not completely define it.

#### 4.2.3. Idiom Literal Divergence

The second topic feature we used is the divergence between the topic distributions of idiomatic samples and literal samples of a given expression. If the topics of an expression's idiomatic samples are clearly separated from the topics of its literal usages, then that expression will have a higher distributional divergence between idiomatic topics and literal topics. In other words, this feature measures the topical drift between the idiomatic and literal sense of an expression and reflects how well a topic model would do at discriminating idiomatic and literal usage. Similarly to TDS, we trained an LSI topic model and reported the KL-divergence between the topic distribution of idiomatic samples and literal samples for each of the 26 expressions in the *ILD* column of Table 2. We calculated its correlation with Shapley values and, interestingly, found a very weak negative correlation (−0.1036). This indicates that the usefulness of an idiomatic expression in training is not due to the difference between topic distributions of its idiomatic usages and literal usages. If anything, the negative correlation implies that idioms with a higher topical divergence are less useful, whereas idioms with a low divergence (i.e., similar topics in both literal and idiomatic usage) are more useful.

#### 4.2.4. Dataset Properties

Across the expressions in our dataset there is variation both in terms of the number of examples and the percentage of idiomatic examples. Consequently, it may be that the Shapley value of an expression is dependent on these data properties, rather than on the idiomatic properties of the expressions. To explore this possibility we first calculated the correlation between the size of the training set (*Size* column of Table 2) for each idiomatic expression and its Shapley values, and then calculated the correlation between the Shapley values of the expressions and the percentage of idiomatic usages (*Ratio* column of Table 2). These correlations are very weak (0.1711 and 0.0235), indicating that usefulness is a reflection neither of dataset size, nor the percentage of idiomatic usage in the sample.

### 4.3. Generalizability of the Model

An important property of a general idiom token identification model is its ability to distinguish idiomatic usage from literal usage of idiomatic expressions which are not seen while training the model. Here, an interesting question is what is the minimum number of idiomatic expressions needed for model training to attain generalizability. Adding more idiomatic expressions will improve performance, but how quickly the model attains good performance on unseen idiomatic expressions (expressions which are not included in the training set) depends on the specific idiomatic expressions included in training. We use the already computed Shapley values, as well as other features, to select idiomatic expressions for training and analyse how quickly the model attains good generalizability.

In this experiment, we first divided the list of 26 idiomatic expressions into 5 folds and considered expressions from 4 folds for training (*train-expressions*) and expressions from the remaining fold (*test-expressions*) for evaluation. From the list of *train-expressions* we chose the best *k* expressions by using different strategies. We then trained a general idiom token identification model using all the samples with these *k* expressions and evaluated on samples with *test-expressions*. We repeated this experiment for different values of *k* and analysed how quickly the models attain good generalizabilty as the value of *k* increases.

The first strategy for selecting best *k* expressions is by using their Shapley values. In this strategy we select *k* expressions with the highest Shapley value, i.e., the most useful *k* expressions. If the Shapley value truly reflects the usefulness of expressions, then this strategy will attain good generalizability with a small value of *k*. By way of this strategy we also validate the computed Shapley value analysis.

We also used strategies based on the other potentially useful features. In these strategies we select *k* expressions that have the highest fixedness score, topic divergence similarity (TDS), idiom literal divergence (ILD), and size respectively. This allows us to compare the impact of these features: if a strategy based on feature A attains good generalizability quicker than a strategy based on feature B, this indicates that feature A contributes to the usefulness of the expressions more than feature B does. We also employ a random selection strategy where we randomly choose *k* expressions, acting as a baseline in this experiment.

**Figure 1:**
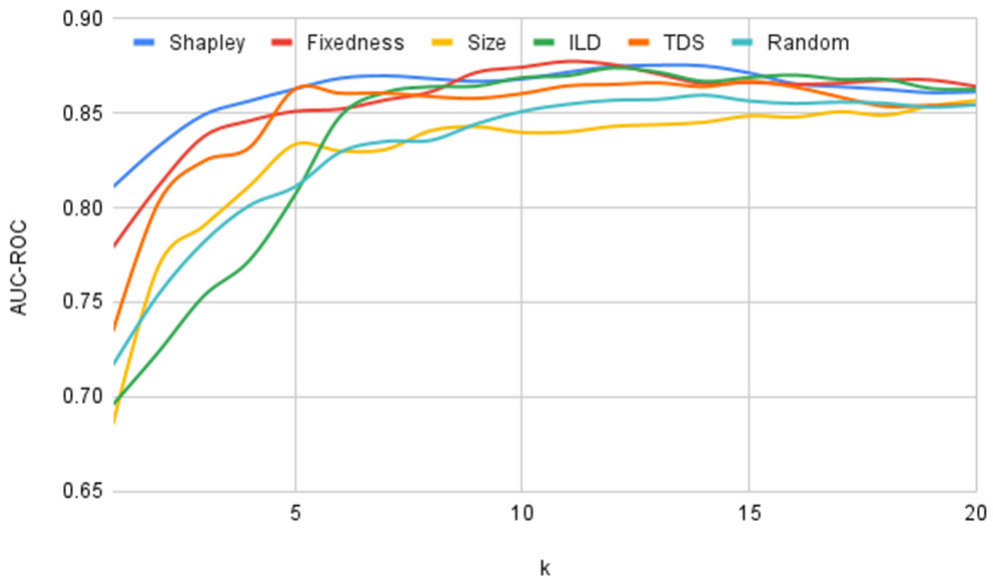
Changes in performance on unseen expressions by varying number of training expressions.

For each selection strategy and for each value of *k* we trained and evaluated the model using 5 versions of *train-expressions* and *test-expressions* similar to a standard 5-fold cross validation method. We further repeated this procedure 10 times with different divisions of the 26 idiomatic expressions into 5 folds and plotted the average values of AUC-ROC score for each selection strategy and for each value of *k* in Figure 1. If the curve corresponding to one selection strategy is above the curve corresponding to another selection strategy, this indicates that this strategy is better at attaining good generalizability.

**Figure 2:**
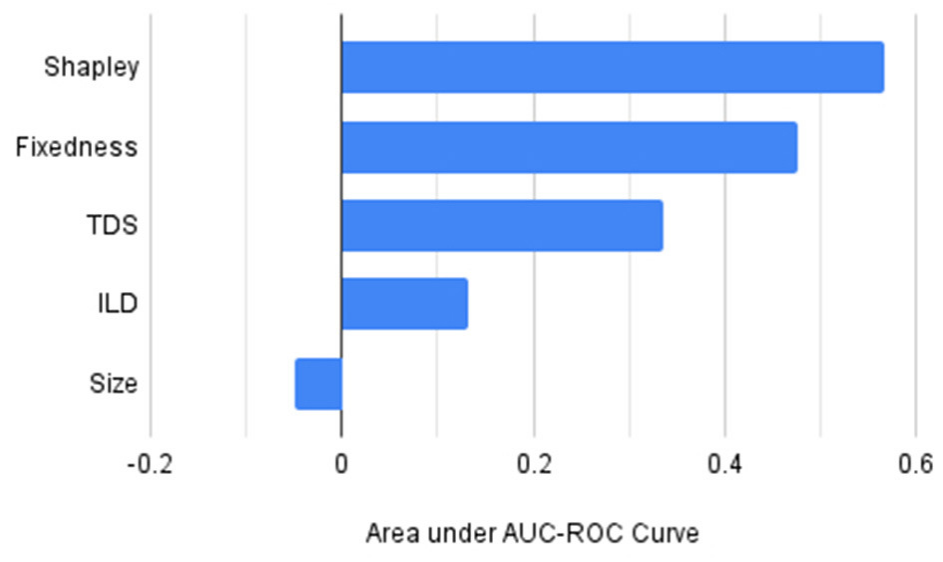
Area under the performance plot on unseen expression by using different selection strategies after subtracting the randomized basline performance.

For comparing the generalizability of different selection strategies we calculated the area under the performance plot shown in Figure 1 and presented in Table 3. Furthermore, to highlight the relative performance of each of these orderings with respect to the random baseline we subtracted the area under the plot for the random selection strategy from the areas for each of the other strategies and charted the results in Figure 2. From Figures 1, 2, and Table 3 it is clear that the Shapely value-based selection strategy is the best selection strategy for generalizability. It is then followed by fixedness and TDS selection strategy, supporting the findings of the correlation study in 4.2 that these two features contribute to an expression's usefulness (recall that fixedness has a correlation of 0.2020 with Shapley, and TDS had a correlation of 0.3359 with Shapley).

**Table 3:** Area under the plot with different selection strategies.

**Selection strategy**	**Area under plot**
Shapley	16.8260
Fixedness	16.7343
TDS	16.5950
ILD	16.3898
Random	16.2578
Size	16.2075

## 5. Conclusion

In our initial experiments we observed that BERT outperforms Skip-Thought embeddings. We suspect that the better performance of BERT on idiom token identification is due to BERT embeddings amplifying the signal of contextual incongruity more than Skip-Thought vectors do. Furthermore, we performed a sequence of experiments exploring performance-based metrics as proxies for idiom usefulness, as well as exploring a range of other factors to analyse in more detail the type of information the model finds useful.

We have found that using a Shapley value calculation provides a very good estimate of a given expression's usefulness on a general idiom identification task and reveals which idioms are most useful for inclusion in the training set. In fact, when using Shapley ordering as few as 7 expressions are sufficient to attain generalizability (as evidenced by an AUC of 0.87 on unseen expressions). And though this is an exciting finding, it should be pointed out that what we consider the usefulness of an idiom is just as much a reflection of the usefulness of the training sample that contains it.

In trying to understand what features actually make a given expression more or less useful, we have explored fixedness as an idiom-intrinsic property, as well as topic-based properties. It is interesting that in our analysis we found that TDS rather than ILD was consistently a stronger indicator of expression usefulness for learning the task of general idiom token identification. This is slightly surprising as one might expect that providing a model with training data where on an expression by expression basis there is a large divergence between the topic distributions of the idiomatic and literal samples would be the most direct way to enable the model to learn that the topic in which an expression is used is a signal for idiomatic versus literal usage. This intuition would suggest that ILD should be a stronger predictor of usefulness than it is. Instead, we found that providing training data that maximizes coverage across topics (as measured by TDS) is a more useful form of topic information. However, more generally our correlation study between different features of idiomatic expressions, and also our generalizability study, has shown that there is no one dominant property that makes an expression useful, but rather both fixedness and topic features in combination contribute to an expression's usefulness. This finding speaks to the complexity of idiomatic usage in language, and suggests that BERT state-of-the-art performance on the task of general idiom token identification is attributable to its ability to combine multiple forms of information (syntactic, topic, and so on) rather than, as was the case in prior work on idiom token identification, to focus on a specific information type as the explanatory signal for idiomatic usage behavior across all expressions.

## Data Availability Statement

The original contributions presented in the study are included in the article/supplementary material, further inquiries can be directed to the corresponding author/s.

## Author Contributions

VN, FK, and JK: conceptualized and designed the study, contributed to the analysis of the results, and the writing of the manuscript. VN: prepared the data and implemented, executed the experiments, and finalized the manuscript. All authors contributed to the critical revision and read and approved the final manuscript.

## Funding

This research was conducted with the financial support of Science Foundation Ireland under Grant Agreement No. 13/RC/2106_P2 at the ADAPT SFI Research Centre at Technological University Dublin. ADAPT, the SFI Research Centre for AI-Driven Digital Content Technology, is funded by Science Foundation Ireland through the SFI Research Centres Programme.

## Conflict of Interest

The authors declare that the research was conducted in the absence of any commercial or financial relationships that could be construed as a potential conflict of interest.

## Publisher's Note

All claims expressed in this article are solely those of the authors and do not necessarily represent those of their affiliated organizations, or those of the publisher, the editors and the reviewers. Any product that may be evaluated in this article, or claim that may be made by its manufacturer, is not guaranteed or endorsed by the publisher.

## Footnotes

^1^12-layer, 768-hidden, 12-heads, and 110M parameters, trained on lower-cased English text.

^2^The scikit-learn library (Pedregosa et al., 2011) is used for training the MLP classifier with the Adam solver (Kingma and Ba, 2014) with learning rate 0.001. We trained the model with a maximum of 200 epochs. Note that the embedding of the sentences into vector representations is done using off-the shelf BERT embeddings. Consequently, the training aspect of our experiments is restricted to just the MLP classifier which has around 77K parameters, so the model training is not complex. The time taken for any of our training instances was less than 2 min on a Macbook Pro machine. All our experimentation codes are available in https://github.com/vasudev2020/Shapley.

^3^ https://people.eng.unimelb.edu.au/paulcook/English_VNC_Cook.zip

^4^Using the gensim library https://radimrehurek.com/gensim/.
